# The Quality-of-Life Implications of Helplessness in Middle-Age and Older Nigerians Living With HIV

**DOI:** 10.1093/geroni/igaf122.3862

**Published:** 2025-12-31

**Authors:** Victor Elisha, Tochukwu Okolie, Anthony Briggs, Chinelo Nduka, Candidus Nwakasi

**Affiliations:** University of Connecticut, Farmington, Farmington, Connecticut, United States; University of Connecticut, Mansfield Center, Connecticut, United States; NYU Grossman School of Medicine, New York, New York, United States; Nnamdi Azikiwe Teaching Hospital, Nnewi, Anambra, Nigeria; University of Connecticut, Storrs, Connecticut, United States

## Abstract

**Background:**

In 2023, the estimated HIV prevalence in Nigeria was 2.1%, ∼ 2 million people living with HIV (PLWH). While interventions have improved physical health and reduced mortality among PLWH in Nigeria, limited research has examined the impact of perceived helplessness on their overall wellbeing. This study investigated the effect of helplessness on wellbeing by focusing on health-related quality-of-life (HRQoL), subjective health status (SHS), and subjective cognitive decline (SCD).

**Methods:**

We recruited 150 PLWH (mean age = 55.2 years) to complete a questionnaire assessing social, psychosocial, and health factors. We modeled whether perceived helplessness was associated with physical-HRQoL (P-HRQoL) and mental-HRQoL (M-HRQoL), SHS, and SCD.

**Results:**

Higher perceived helplessness was associated with lower physical health-related quality of life (P-HRQoL; β = -0.275, p < 0.001) and mental health-related quality of life (M-HRQoL; β = -0.180, p < 0.001). It was also associated with subjective health (β = 0.010, p < 0.001) and higher report of SCD (β = 0.443, p < 0.001. Increased age was associated with lower P-HRQoL, M-HRQoL, SHS, and higher SCD. Additionally, more years since HIV diagnosis was associated with lower physical and mental health-related quality of life.

**Discussion:**

While medical advancements and public health efforts improve longevity among PLWH, many PLWH in Nigeria face persistent stressors from HIV-related stigma, and social disadvantages. These may contribute to perceived helplessness, and a diminished sense of control, thus impacting their wellbeing. Our findings underscore the importance of developing psychosocial interventions for middle-age and older PLWH in Nigeria

